# In Silico Studies on GCP-Lys-OMe as a Potential 14-3-3σ Homodimer Stabilizer

**DOI:** 10.3390/ph15101290

**Published:** 2022-10-20

**Authors:** Ghazi Aljabal, Beow Keat Yap

**Affiliations:** School of Pharmaceutical Sciences, Universiti Sains Malaysia, Gelugor 11800, Penang, Malaysia

**Keywords:** 14-3-3 sigma, dimer, stabilizer, cancer, docking, molecular dynamics

## Abstract

14-3-3 sigma is a vital negative cell cycle regulator. Its expression is consistently downregulated in many types of cancer through gene promoter hypermethylation or proteasomal degradation. 14-3-3 sigma needs to form a homodimer to be functional, while dimers are less prone to degradation than monomers. This suggests that a homodimer stabilizer may increase the tumor suppressive activities of 14-3-3 sigma. However, no known homodimer stabilizer of 14-3-3 sigma has been reported to date. Therefore, this study attempts to test the potential capability of GCP-Lys-OMe (previously reported to bind at the dimer interface of 14-3-3 zeta isoform), to bind and stabilize the 14-3-3 sigma homodimer. In silico docking of GCP-Lys-OMe on 14-3-3 sigma showed more favorable interaction energy (−9.63 kcal/mole) to the dimer interface than 14-3-3 zeta (−7.73 kcal/mole). Subsequent 100 ns molecular dynamics simulation of the GCP-Lys-OMe/14-3-3 sigma complex revealed a highly stable interaction with an average root-mean-square deviation of 0.39 nm (protein backbone) and 0.77 nm (ligand atoms). More contacts between residues at the homodimer interface and a smaller coverage of conformational space of protein atoms were detected for the bound form than for the apo form. These results suggest that GCP-Lys-OMe is a potential homodimer stabilizer of 14-3-3 sigma.

## 1. Introduction

The 14-3-3 proteins are a family of highly conserved regulatory mammalian proteins that are expressed in all eukaryotic cells [1,2,3]. Seven human 14-3-3 isoforms, β-beta, ε-epsilon, γ-gamma, η-eta, σ-sigma, τ-tau and ζ-zeta, with more than 500 protein partners, have been identified in mammalian cells [4,5,6,7,8]. Consistent with that, 14-3-3 proteins are well-known to be important for various cellular processes, including cell proliferation, cell cycle control and cell apoptosis [9,10,11,12,13,14]. Despite the dimeric structural similarity among the mammalian 14-3-3 isoforms, many reports have shown isoform-specific interactions with 14-3-3 binding partners [15,16,17]. It is believed that the difference in the residues of the region adjacent to the amphipathic groove and the unconserved residues forming the outer surface of 14-3-3 proteins play a key role in 14-3-3 specific binding targets [18,19]. In addition, reports have indicated that the N-terminal as well as the position and angles between the monomers vary among 14-3-3 isoforms, which confer an isoform-specific partner interaction [18,20,21]. Besides the difference in the structure, 14-3-3 isoforms also exhibit distinct expression and distribution patterns. For instance, while most 14-3-3 isoforms are upregulated in many cancers, 14-3-3σ is consistently downregulated in numerous cancer types. Furthermore, 14-3-3σ is mainly localized in the cytoplasm, whereas 14-3-3ζ is mainly present in the nucleus [22].

Previous studies have identified 14-3-3σ as a tumor suppressor as it plays a pivotal role in controlling tumor metabolic reprogramming and thus cancer cell growth and metastasis [23]. For example, it has been reported that 14-3-3σ protects P53 against MDM2-mediated ubiquitination and degradation, resulting in direct control of the G2-M checkpoint of the cell cycle [24,25,26]. In addition, 14-3-3σ is also involved in cell cycle arrest regulation via acting as a cyclin-dependent kinase (Cdk) inhibitor [27,28]. Moreover, 14-3-3σ was also found to protect the cells against Akt-mediated tumorigenesis via negatively regulating the oncogenic activity of the protein kinase B [25]. Furthermore, studies have shown that 14-3-3σ can control cell proliferation and protect against cancer metastasis via regulating the nuclear export of the p65 subunit of the NF-ĸB transcription factor [29,30]. On top of that, 14-3-3σ has also been reported to regulate the expression of the human TASK-3 channel and the oncogenic activity of transcriptional coactivator TAZ, which results in controlling cancer cell proliferation and migration [31,32,33,34]. These observations indicate that 14-3-3σ is a potentially important pharmacological target in cancer. Consistent with these observations, the low expression level of 14-3-3σ has been reported in many types of cancer, which has been linked to either the promoter hypermethylation of its gene (Sfn) or direct 14-3-3σ degradation through ubiquitination, which eventually aborts its normal physiological role against tumor growth and metastasis [35,36,37,38,39]. 

The 14-3-3σ protein isoform is the most unique member of the 14-3-3 family as it is the only isoform that only exists as a homodimer while other isoforms are expressed as both homo- and heterodimers [40,41,42]. Although 14-3-3σ generally shares the same structural shape as the other isoforms, i.e., a cup-like shape with nine elongated bundles of anti-parallel helices in each monomer (with H1–H4 forming the dimer interface and H5–H9 forming the amphipathic ligand-binding groove), only two key residues involved in the dimerization of 14-3-3σ (Leu12 and Tyr84) are highly conserved across isoforms, while the remaining five residues (i.e., Ser5, Glu20, Phe25, Gln55 and Glu80) are only conserved across different species of 14-3-3σ [18,43]. Importantly, mutation of these residues has been reported to result in the dissociation of the dimeric form of 14-3-3σ and diminish its function [18,43,44]. As the homodimer form is crucial for the full activity of 14-3-3σ, a homodimer stabilizer may be potentially useful in increasing the activity of 14-3-3σ [45,46,47,48]. A homodimer stabilizer may also be potentially useful to address the downregulation of 14-3-3σ in cancer. Although there is no direct evidence showing the direct correlation between 14-3-3σ downregulation and homodimer dissociation, it has been previously reported that a monomer is more prone to degradation than a dimer [49,50]. This led us to hypothesize that having a compound such as GCP-Lys-OMe to stabilize the 14-3-3σ homodimer will not only decrease the likelihood of 14-3-3σ dissociating into a monomer and becoming functionally inactive but also decreases its tendency to be degraded.

Unfortunately, to date, no ligand has been reported to target the central cavity of the dimer interface of the 14-3-3σ and stabilize its homodimeric form, as the reported modulators of 14-3-3σ (both stabilizers and inhibitors) generally target the amphipathic ligand-binding groove [23,51]. Nevertheless, one ligand (GCP-Lys-OMe) has been recently reported to bind to the dimer interface of the 14-3-3ζ isoform [47], though it is not known if the compound can stabilize the 14-3-3ζ dimer. GCP-Lys-OMe is a simple derivative of the polycationic guanidiniocarbonyl-pyrrole (GCP), which was developed by Schmuck and co-workers in 1999 [52] as a recognition motif for oxoanions such as carboxylates. The GCP moiety is unique in that it can bind to carboxylate even in a competitive aqueous media containing ions and salts, making GCP a promising candidate for protein recognition [52,53]. Previously, GCP-based multivalent ligands with a central benzene ring or lysine residues have been reported as a specific stabilizer of 14-3-3ζ/C-Raf and 14-3-3ζ/Tau PPIs [54,55]. Another ligand that was found to be able to stabilize the 14-3-3ζ dimer is fragment 2, but the exact binding site of the ligand on 14-3-3ζ was not resolved experimentally [48].

Therefore, in this study, we sought to investigate the ability of one of the reported compounds, i.e., GCP-Lys-OMe, to (1) bind at the homodimer interface of 14-3-3σ and (2) stabilize the 14-3-3σ homodimer. Different in silico techniques, such as docking, molecular dynamics simulations and various analysis tools are used in this study to address these questions. To the best of our knowledge, this is the first study that attempts to use an in silico technique to predict the effect of a ligand on the stability of the 14-3-3σ homodimer.

## 2. Results and Discussion

### 2.1. The Cavity at the Dimer Interface of 14-3-3σ as a Putative Druggable Pocket

To investigate the potential druggable site(s) of 14-3-3σ, the X-ray coordinate of the 14-3-3σ protein (PDB ID: 1YZ5) was submitted to the FTmap web server (https://ftmap.bu.edu, accessed on 16 April 2022). In general, FTmap [56] uses a library of 16 small organic probe molecules with differing sizes, shapes and polarities to scan the entire surface of the protein and find the most favorable position for each probe. These probes are then clustered and ranked based on their average energy. Regions on the protein surface that bind to different types of these probes are called the consensus sites, or the binding hot spots.

**Figure 1:**
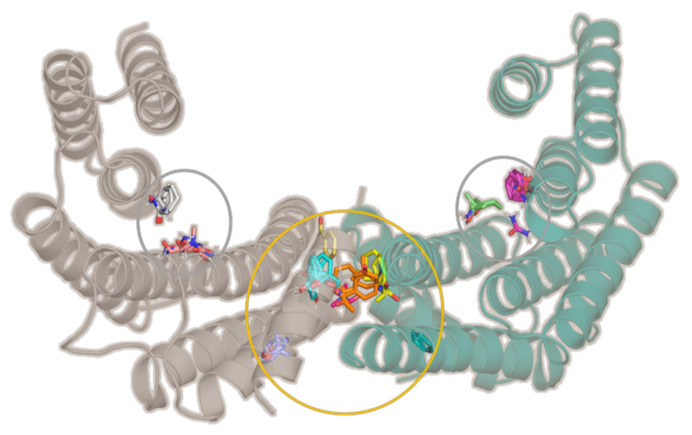
The X-ray crystal structure of 14-3-3σ dimer (PDB ID: 1YZ5). Two amphipathic ligand-binding pockets (gray circles) and a cavity at the homodimer interface (orange circle) were identified by FTMap as the binding hot spots.

As expected, the amphipathic ligand-binding pockets, which are well-known to be involved in 14-3-3σ’s interaction with various protein partners, were identified as one of the key binding hot spots (Figure 1, Figure S1, Supplementary Materials), whereas at the homodimer interface, the cavity at the center of the homodimer interface was found to be the most druggable, which is in agreement with previous observation for GCP-Lys-OMe on 14-3-3ζ isoform [47].

### 2.2. GCP-Lys-OMe Can Bind to 14-3-3σ at the Homodimer Interface

**Figure 2:**
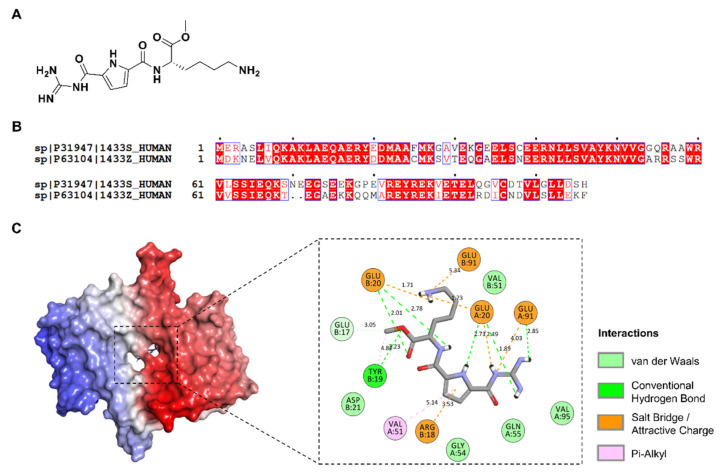
(**A**) Chemical structure of GCP-Lys-OMe. (**B**) Sequence alignment of the dimer interface helices (H1–H4) of the human 14-3-3σ and human 14-3-3ζ isoforms (UniProtKB codes, human 14-3-3σ: P31947; human 14-3-3ζ: P63104 as performed with ClustalW and ESPript 3.0, with the red box, white character: strict identity; red character: similarity in the group; blue frame: similarity across the group. (**C**) Docking of GCP-Lys-OMe against 14-3-3σ. (Left) The protein is represented as the red- and blue-colored surface for each monomer, respectively, while the docked compound (lowest binding energy conformation, in the stick representation) is indicated by a black box. (Right) Two-dimensional (2D) interaction map showing the interactions between GCP-Lys-OMe and the homodimer interface of 14-3-3σ. The docked compound is shown as a stick with gray-colored carbon while the residues at the homodimer interface of 14-3-3σ protein are shown as discs and colored based on their types of interaction with the compound.

To investigate whether GCP-Lys-OMe (Figure 2A) (which was previously shown to target the dimer interface of 14-3-3ζ) is also able to bind to the homodimer interface of 14-3-3σ, the compound was subjected to docking studies via AutoDock 4.2. As a control, the compound was first docked against the 14-3-3ζ protein before the same parameters were used for docking the compound against the 14-3-3σ protein. Blind docking on 14-3-3ζ protein revealed that GCP-Lys-OMe favors the dimer interface over the amphipathic ligand-binding pocket and other potential binding sites of the 14-3-3ζ, whilst focus docking of the compound at the dimer interface of 14-3-3ζ showed that GCP-Lys-OMe interacts with both Asp20A and Asp20B and Tyr19A, Arg18A and Ser58B of 14-3-3ζ (Figure S2, Supplementary Materials). These observations are in agreement with the findings reported by Ehlers et al. [47].

When GCP-Lys-OMe was docked against the whole structure of the 14-3-3σ protein, the compound was also found to favor the dimer interface of 14-3-3σ, although both isoforms only share 65% sequence identity at the dimer interface region (H1–H4) (Figure 2B). Thus, only 69 out of 106 residues have the same amino acid in the same positions in the aligned sequence. Additionally, two of the five key residues reported to be important for the interaction between GCP-Lys-OMe and 14-3-3ζ [29], i.e., Asp20 and Ser58, are not observed in 14-3-3σ, as the corresponding positions are occupied by Glu20 and Ala58, respectively, in the aligned sequence. Intriguingly, focus docking of GCP-Lys-OMe at the dimer interface of the 14-3-3σ protein (Figure 2C) showed stronger binding to the 14-3-3σ protein than that of 14-3-3ζ, with estimated free binding energies of −9.63 kcal/mol and −7.73 kcal/mol, respectively. A comparison of the docked conformation of GCP-Lys-OMe on 14-3-3σ (Figure 2C) versus 14-3-3ζ (Figure S2, Supplementary Materials) revealed additional interactions (electrostatic and hydrogen bonds) between GCP-Lys-OMe and both Glu91A and Glu91B of 14-3-3σ. This suggests that GCP-Lys-OMe is a potential binder of the 14-3-3σ protein at the homodimer interface.

**Figure 3:**
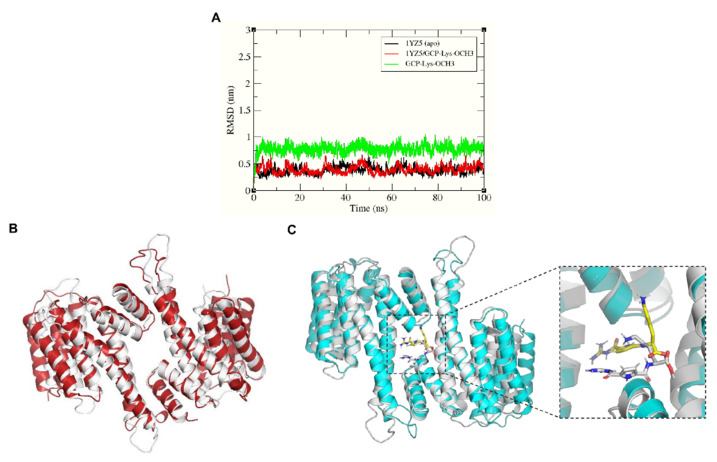
(**A**) The RMSD plots of the protein and ligand backbone atoms for the simulated systems of the apo form of 14-3-3σ (PDB: 1YZ5) and the 14-3-3σ/GCP-Lys-OMe complex. Superimposition of the initial conformation (white ribbon) and 100 ns conformation of the 14-3-3σ protein of (**B**) the apo system (red ribbon) and (**C**) the 14-3-3σ/GCP-Lys-OMe system (cyan ribbon). The corresponding conformations of the compound GCP-Lys-OMe initially and at 100 ns are shown in white and yellow sticks (inlet), respectively.

To further confirm the binding of GCP-Lys-OMe at the dimer interface of 14-3-3σ, the stability of the docked complex was investigated. A 100 ns MD simulation was performed using GROMACS 2016 for both the ligand-free (apo) 14-3-3σ protein and the 14-3-3σ/GCP-Lys-OMe complex. The root-mean-square deviation (RMSD) of the protein and ligand backbone atoms was calculated for each system to determine their structural stability. Figure 3A clearly shows that both the apo and the ligand-bound systems were highly stable throughout the simulation time, with average protein backbone RMSD values of 0.38 and 0.39 nm, respectively. Similarly, the backbone atoms of GCP-Lys-OMe were also stable throughout the simulation time with an average RMSD value of 0.77 nm. 

Superimposition of the initial (0 ns) and the final (100 ns) configurations of the simulated systems (Figure 3B,C) showed that the helices at the dimer interface (H1–H4) of the protein were in near complete alignment in both systems, thus highlighting the stability of the protein backbone at the dimer interface throughout the simulation time for both the apo and bound systems. This also indicates that the binding of GCP-Lys-OMe at the dimer interface did not negatively affect the stability of the homodimer interface of 14-3-3σ. The helices at the C-terminus (H5-H9) of the protein, however, did not superimpose very well between the final configuration and the initial configuration for either the apo or the bound systems. This is not surprising as it has been reported previously that H5-H9 tend to change their conformation between a closed state and an open state [57]. A previous study by Liu et al. [44] demonstrated that 14-3-3 proteins may bind to target proteins through the transition between open and closed conformations, which was also confirmed later by Hu et al. via molecular dynamics simulations [57].

The initial (docked) conformation of GCP-Lys-OMe is also significantly different from the final conformation. The major change in conformation, however, was predominantly observed only during the first 5 ns of simulation, after which GCP-Lys-OMe began to adopt a similar, more stable conformation (i.e., a T shape-like conformation where the Lys moiety is at the surface of the dimer interface while the GCP moiety is buried inside the cavity) until the end of the simulation (Figure 3C and Figure S3, Supplementary Materials).

### 2.3. GCP-Lys-OMe Stabilizes 14-3-3σ Homodimer

**Figure 4:**
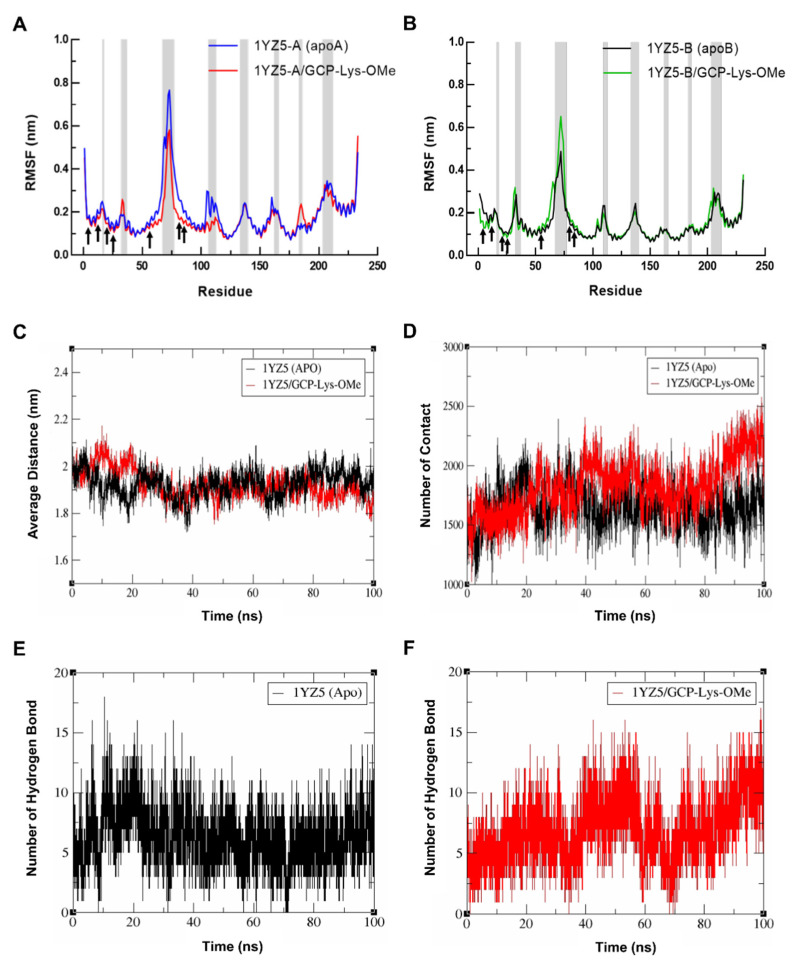
Root-mean-square fluctuation (RMSF) of the Cα atoms versus residue number for (**A**) monomer A and (**B**) monomer B of 14-3-3σ for the simulated systems of the apo form of 14-3-3σ and the 14-3-3σ/GCP-Lys-OMe complex. Loop regions are indicated by gray shades. (**C**) Plots of the distance between the mass center of the helices H1 to H4 from both monomers of 14-3-3σ in the apo (black) and GCP-Lys-OMe-bound complex (red). (**D**) Changes in the number of contacts between the helices H1 to H4 from both monomers of 14-3-3σ in the apo (black) and GCP-Lys-OMe-bound complex (red). Hydrogen bond profile of the dimer interface residues of the apo 14-3-3σ (**E**) and 14-3-3σ/GCP-Lys-OMe (**F**) systems.

To investigate the ability of GCP-Lys-OMe to stabilize the 14-3-3σ homodimer, the residual flexibility and the conformational change in 14-3-3σ residues in both the apo and GCP-Lys-OMe-bound systems during the 100 ns simulation were compared. The root-mean-square fluctuation (RMSF) was measured for the Cα atoms of both monomers of 14-3-3σ in both the apo and GCP-Lys-OMe-bound systems, as illustrated in Figure 4A,B. Both simulated systems shared similar major characteristics where the residual fluctuations were lower for residues in the helices compared to those at the loops where higher RMSF values were recorded. Intriguingly, at the dimer interface, the average RMSF values of the seven key residues from both monomers of the 14-3-3σ protein were found to be modestly lower in the GCP-Lys-OMe-bound system than in the apo form. For example, Ser5, Leu12, Glu20 and Phe25 had modestly lower RMSF values in both monomer A and B of the GCP-Lys-OMe-bound 14-3-3σ protein, while the RMSF values of Gln55, Glu80 and Tyr84 were significantly lower in monomer A but slightly higher in monomer B. These observations indicate that the binding of GCP-Lys-OMe to the dimer cavity somewhat decreases the flexibility of the residues at the dimer interface. 

To further probe the impact of the GCP-Lys-OMe ligand on the stabilization of the 14-3-3σ homodimer, the average distance between the mass center of helices H1–H4 from both monomers was calculated from the MD trajectories. Figure 4C shows that the average distance between H1–H4 of the two monomers was significantly higher in the ligand-bound system in the first 20 ns, presumably due to the system equilibrating to accommodate the ligand at the dimer interface. The average distance, however, started to normalize thereafter with a similar fluctuation in distance between the apo and ligand-bound systems of around 1.92 nm and 1.90 nm, respectively, until about 80 ns, where the average distance for the ligand-bound system began to fluctuate at a lower value compared to the apo form. At 100 ns of the simulation time, the average distance between H1–H4 of both monomers of the 14-3-3σ dimer for the apo and the ligand-bound system was approximately 1.91 nm and 1.81 nm, respectively. 

To understand the cause for the decrease in the average distance between the monomers in the last 20 ns of the simulations of the ligand-bound system, the number of contacts between H1–H4 from the two monomers of 14-3-3σ was extracted from the same MD trajectories using the gmx mindist tool. In general, a contact between any pair of atoms of H1–H4 residues between the two monomers that is within 0.6 nm is counted as one contact. Figure 4D clearly shows that the change in the number of contacts between the two monomers at the dimer interface of 14-3-3σ is highly consistent with the distance fluctuation observed, with an obvious increase in contacts in the ligand-bound system after 80 ns of simulations. At 100 ns of the simulation time, the number of contacts between the two monomers (H1–H4) for the apo and the ligand-bound forms was 1809 and 2260, respectively. These observations highlight the ability of GCP-Lys-OMe to enhance the interactions between the monomers of 14-3-3σ and subsequently stabilize the homodimer form of 14-3-3σ. 

Further analysis of the hydrogen bond profiles of the residues at H1–H4 (the dimer interface) of 14-3-3σ (apo) (Figure 4E) and 14-3-3σ/GCP-Lys-OMe systems (Figure 4F) revealed that the increase in contact in the ligand-bound system was likely to be driven by the additional intermolecular hydrogen bonds involving the residues at the dimer interface, as very similar analysis profiles between the number of hydrogen bonds and the number of contacts were observed in both systems. For example, the number of hydrogen bonds in the ligand-bound system also showed an increasing trend 80 ns into simulations, with the number of hydrogen bonds at 100 ns being significantly higher in the ligand-bound system (13 hydrogen bonds) than in the apo 14-3-3σ system (8 hydrogen bonds) (Table S1, Supplementary Materials). Consistent with this observation, the number of residue pairs between monomers with hydrogen bond occupancy ≥ 10% throughout the 100 ns simulations was also higher in the ligand-bound system (23 pairs) than in the apo 14-3-3σ system (19 pairs) (Tables S2 and S3, Supplementary Materials). 

It is also worth noting that the increase in the number of contacts was not limited to direct contact between monomers. GCP-Lys-OMe also formed hydrogen bonds, electrostatic interactions and van der Waals interactions with other residues from both monomers throughout the simulations (Figure S3, Supplementary Materials). For example, Glu20B was found to form a hydrogen bond with GCP-Lys-OMe for 61.8% of the total simulation time (Table S4, Supplementary Materials). Such indirect contact between monomers further enhances the stability of the homodimer.

**Figure 5:**
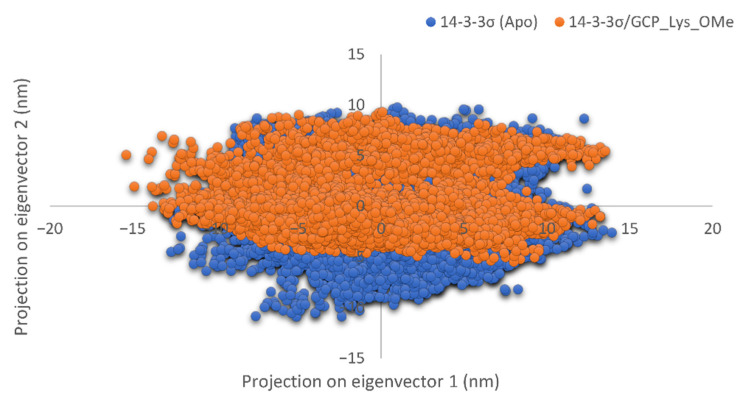
Projection of motion of protein atoms along the first two dominant eigenvectors (PC1 and PC2) of the apo 14-3-3σ (blue) and 14-3-3σ/GCP-Lys-OMe complex systems (orange).

To further validate the effect of GCP-Lys-OMe on the stability of the 14-3-3σ homodimer, principal component analysis (PCA) was performed for both apo and ligand-bound systems using g_covar and g_anaeig tools in GROMACS. The collective dynamic motion and behavior of the atoms in the simulated systems were analyzed using the MD trajectories projected on principal components (PC1 and PC2). Notably, the trajectories of 14-3-3σ (apo) showed higher space magnitudes and covered slightly wider conformational space than the 14-3-3σ/GCP-Lys-OMe complex (Figure 5). The flexibility of the protein in both systems was also analyzed by calculating the trace value for the diagonalized covariance matrix, which is a matrix of eigenvectors and diagonal eigenvalues. The trace values, which are the sums of the eigenvalues, were 75.3212 and 71.1446 nm^2^ for the apo and 14-3-3σ/GCP-Lys-OMe systems, respectively. This indicates that the apo form of 14-3-3σ appeared to cover a slightly larger conformational space than the 14-3-3σ/GCP-Lys-OMe complex due to its greater flexibility. These findings further support the stabilization of the 14-3-3σ protein by GCP-Lys-OMe.

## 3. Materials and Methods

### 3.1. Protein Preparation

The X-ray crystal structures of the apo 14-3-3σ (entry code 1YZ5: resolution 2.8 Å) and 14-3-3ζ (entry code 1QJA: resolution 2.00 Å) were retrieved from the Protein Data Bank (https://www.rcsb.org, accessed on 16 April 2022) to serve as structural models. The crystal structures of 14-3-3σ and 14-3-3ζ were checked for problems related to alternate conformations, missing loops or incomplete residues using the Protein Report tool in *Discovery Studio (DS)*, version 16.1.0.15350; BIOVIA, Dassault Systèmes: San Diego, CA, USA, 2016, and fixed using the Prepare Protein protocol in DS. *AutoDockTools*, version 1.5.6; The Scripps Research Institute: La Jolla, CA, USA, 2009 [58], was used to add polar hydrogens and Kollman charges to the proteins.

### 3.2. Receptor Grid Map Generation

For focus docking where the central cavity at the dimer interface is targeted, a grid box of 70 × 70 × 70 points with a grid spacing of 0.375 Å, centered at the coordinates x: 8.552, y: 28.11 and z: −12.728, was set for 14-3-3σ. On the other hand, a grid box of 70 × 70 × 70 points with a grid spacing of 0.375 Å, centered at the coordinates x: 17.619, y: 4.729 and z: 56.846, was set for 14-3-3ζ. For blind docking, a larger grid box of 126 × 126 × 126 points with a grid spacing of 0.486 Å and the grid box center coordinates of x: 8.172, y: 41.093 and z: 10.343 was generated for 14-3-3σ, while a grid box of 126 × 126 × 126 points with a grid spacing of 0.591 Å and the grid box center coordinates of x: 26.196, y: 4.565 and z: 46.52 was generated for the 14-3-3ζ.

### 3.3. Ligand Preparation

The compound GCP-Lys-OMe was sketched by *ChemDraw Professional*, version 17.1; PerkinElmer Informatics: Waltham, Massachusetts, USA, 2018, minimized by Chem3D, ionized by *Discovery Studio*, version 16.1.0.15350; BIOVIA, Dassault Systèmes: San Diego, CA, USA, 2016, and converted into PDBQT by *AutoDockTools*, version 1.5.6; The Scripps Research Institute: La Jolla, CA, USA, 2009 [58].

### 3.4. Molecular Docking

*AutoDock*, version 4.2; The Scripps Research Institute: La Jolla, CA, USA, 2009 [58] was used for docking the prepared compound according to the grid box coordinates described previously.

### 3.5. Molecular Dynamics Simulations

A total of 100 ns of MD simulation was performed for each system: the apo 14-3-3σ and the 14-3-3σ/GCP-Lys-OMe complex. The *GROMACS*, version 2016.3; University of Groningen, Groningen, Netherlands, 2017, software package with the Gromos96 54a7 force field [59] was used for the nanosecond-scale MD simulation. For the 14-3-3σ/GCP-Lys-OMe complex, the lowest binding energy conformation of GCP-Lys-OMe retrieved from the docking study was used as the initial coordinate (input file) for MD simulation. The topology file of the protein was generated by the GROMACS tool, pdb2gmx, whereas the topology file of the ligand was generated by the PRODRG server (http://davapc1.bioch.dundee.ac.uk/cgi-bin/prodrg, accessed on 4 June 2022). The systems were solvated with the simple-point charge water model (SPC) in a dodecahedron box with a spacing distance of 1 nm around the surface. To neutralize the systems, counter ions were added to balance the charge of the proteins. Then, the systems were minimized using the steepest descent integrator with 50,000 maximum minimization steps and a 0.01 energy step size. The minimized system was then equilibrated in the NVT (constant number of particles, volume and temperature) ensemble for 100 ps at 310 K using the v-rescale coupling method followed by another 100 ps of equilibration in the NPT ensemble (constant number of particles, pressure and temperature) at 1.0 bar using the Berendsen pressure coupling method. After the temperature and pressure equilibrations, MD simulation runs were performed for the models for 100 ns each at 1 bar and 310 K. The short-range non-bonded interactions cut-off was set at 1.2 nm, while long-range electrostatic interactions were treated using the Particle Mesh Ewald (PME) algorithm. The LINCS algorithm was used to constrain the bonds with hydrogen atoms. All simulations were computed with a time step of 2 fs, and the coordinates were recorded every 5000 steps (10 ps) for MD data analysis. Molecular graphic images were produced using the *PyMOL Molecular Graphics System*, version 2.3.3; Schrödinger: LLC, New York, NY, USA, 2019. Graphs were prepared using the *Xmgrace*, version 5.1.25. Grace Development Team: Weizmann Institute of Science, Israel, 2015. All analyses on the MD trajectories, such as the root-mean-square deviation (RMSD), root-mean-square fluctuation (RMSF), minimum distance studies, hydrogen bond analysis and the principal component analysis (PCA), were performed using the analysis tools in *GROMACS*, version 2016.3; University of Groningen, Groningen, Netherlands, 2017.

## 4. Conclusions

In conclusion, we have used in silico molecular docking and MD simulation techniques to investigate the probability of GCP-Lys-OMe targeting the dimer interface of the 14-3-3σ. Molecular docking using *AutoDock*, version 4.2; The Scripps Research Institute: La Jolla, CA, USA, 2009 [58] showed that GCP-Lys-OMe can target the homodimer interface of 14-3-3σ. Further MD simulation studies revealed the stability of the ligand at the cavity of the dimer interface. The ligand was also found to be able to stabilize the 14-3-3σ homodimer by increasing the contact between the monomers. These results encourage the hypothesis that small molecules such as GCP-Lys-OMe can potentially target and stabilize the 14-3-3σ homodimer and that further work to validate the binding and the stabilization effect of GCP-Lys-OMe on the 14-3-3σ homodimer using experimental types of assays such as ligand-binding assays and functional assays would be worthwhile.

## Data Availability

Data is contained within the article and Supplementary Material.

## Supplementary Materials

The following supporting information can be downloaded at: https://www.mdpi.com/article/10.3390/ph15101290/s1. Figure S1: 14-3-3σ binding hotspots by FTMap; Figure S2: Docking of GCP-Lys-OMe against 14-3-3ζ; Figure S3: Snapshots of simulated 14-3-3σ/ GCP-Lys-OMe complex; Table S1: Hydrogen bond pairs between monomers of 14-3-3σ in apo and GCP-Lys-OMe-bound systems at 100 ns simulation; Table S2: Hydrogen bond occupancy between monomers of 14-3-3σ in the apo system throughout the whole 100 ns simulation; Table S3: Hydrogen bond occupancy between monomers of 14-3-3σ in GCP-Lys-OMe-bound systems throughout the whole 100 ns simulation; Table S4: Hydrogen bond occupancy between monomers of 14-3-3σ and GCP-Lys-OMe in GCP-Lys-OMe-bound systems throughout the whole 100 ns simulation.
