# Supplementary material for: In Silico Studies on GCP-Lys-OMe as a Potential 14-3-3σ Homodimer Stabilizer
Source: Pharmaceuticals (Basel). 2022 Oct 20;15(10):1290. doi: 10.3390/ph15101290 (PMC9609495; doi:10.3390/ph15101290)
Supplement: Supplementary file 1 [file pharmaceuticals-15-01290-s001.zip › pharmaceuticals-1948959-supplementary.pdf]

Pharmaceuticals

SUPPLEMENTARY MATERIALS

In Silico Studies on GCP-Lys-OMe as a Potential 14-3-3σ Homodimer Stabilizer

Ghazi Aljabal and Beow Keat Yap *

School of Pharmaceutical Sciences, Universiti Sains Malaysia, Penang 11800, Malaysia

* Correspondence: beowkeat@usm.my; Tel.: +604-653-2207

Table of Content

Figure S1: 14-3-3σ binding hotspots by FTMap

Figure S2: Docking of GCP-Lys-OMe against 14-3-3ζ

Figure S3: Snapshots of simulated 14-3-3σ/ GCP-Lys-OMe complex

Table S1: Hydrogen bond pairs between monomers of 14-3-3σ in apo and GCP-Lys-OMe-bound systems at 100 ns simulation.

Table S2: Hydrogen bond occupancy between monomers of 14-3-3σ in apo system throughout the whole 100 ns simulation.

Table S3: Hydrogen bond occupancy between monomers of 14-3-3σ in GCP-Lys-OMe-bound systems throughout the whole 100 ns simulation.

Table S4: Hydrogen bond occupancy between monomers of 14-3-3σ and GCP-Lys-OMe in GCP-Lys-OMe-bound systems throughout the whole 100 ns simulation.

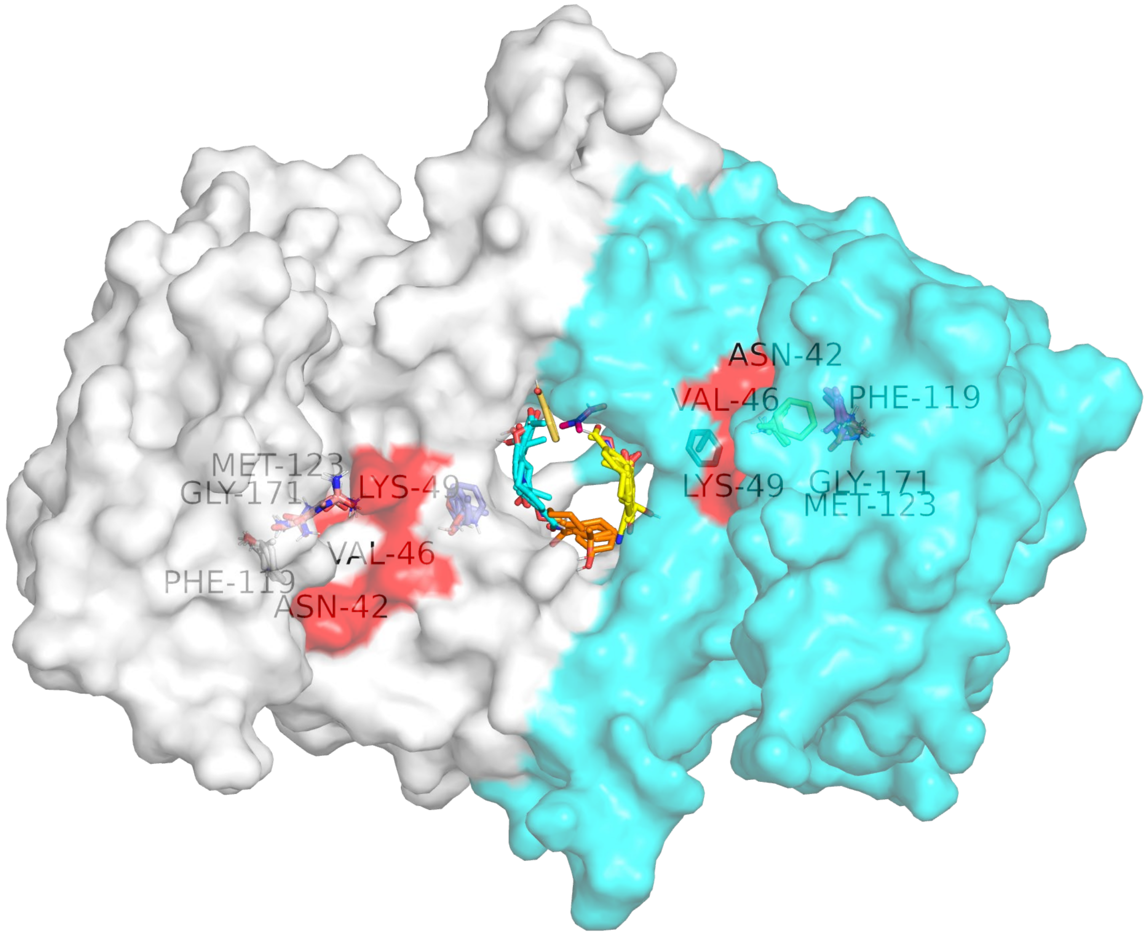

Figure S1. 14-3-3σ binding hotspots by FTMap. Residues lining the amphipathic ligand binding pockets of 14-3-3σ homodimer (white/cyan surface) are labeled, with key interacting residues highlighted in red. The probes (shown in sticks) are mainly bound at the center cavity of the homodimer interface and the amphipathic pockets (monomer A and monomer B) of 14-3-3σ.

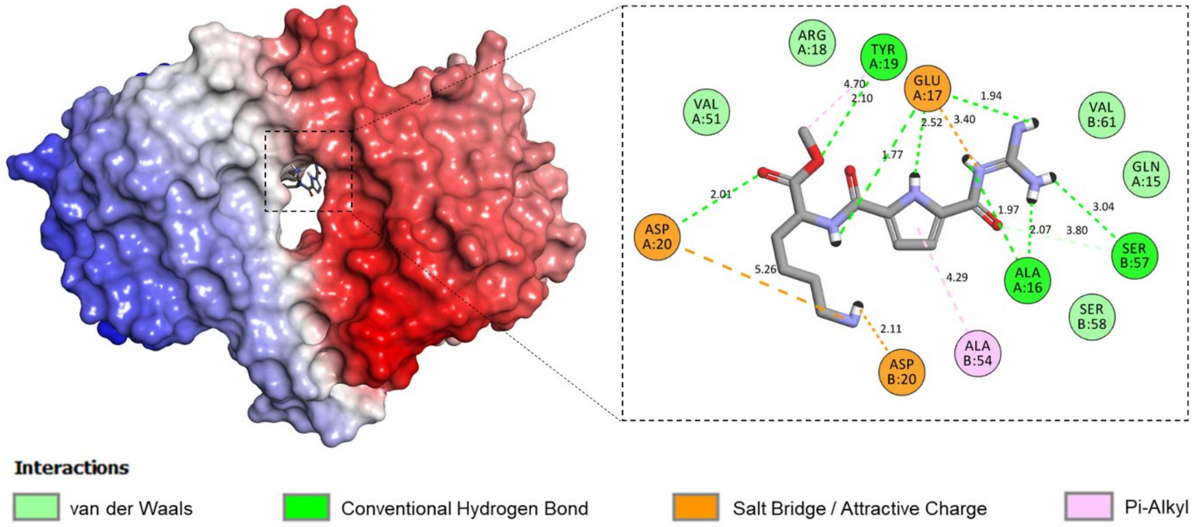

Figure S2. Blind docking of GCP-Lys-OMe against 14-3-3ζ (left). The protein is represented as red and blue colored surface for both monomers, respectively. The lowest energy conformation of the docked compound is indicated in black dashed square and shown as stick. 2D interaction map of the lowest energy conformation from focus docking of GCP-Lys-OMe into the dimer interface of 14-3-3ζ (right, expanded box). The docked compound is shown as stick with carbon colored grey while the dimer interface residues of 14-3-3ζ protein are shown as discs and colored based on their interaction type.

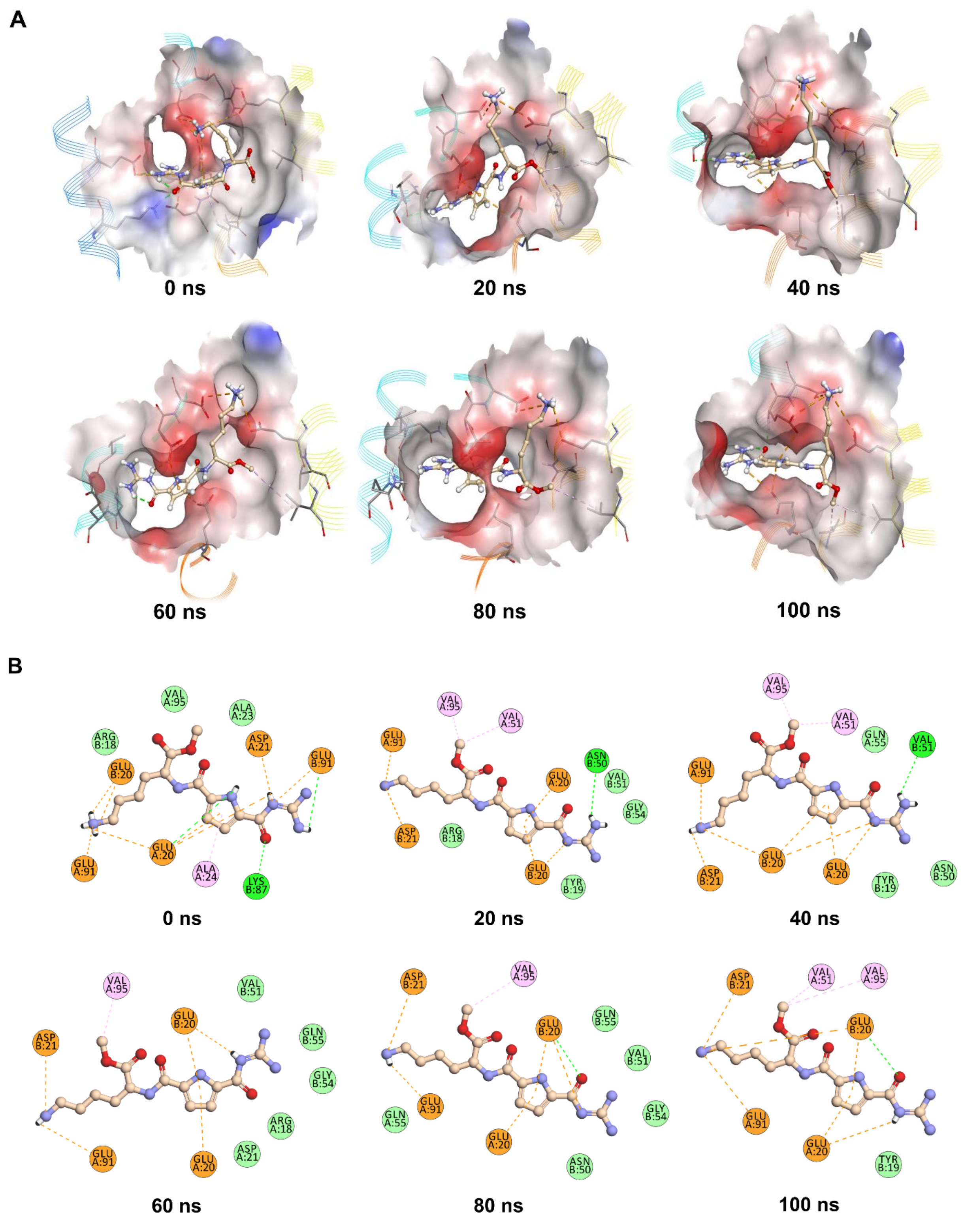

Figure S3. (**A**) Snapshots of the simulated 14-3-3σ/ GCP-Lys-OMe complex at 0 ns, 20 ns, 40 ns, 60 ns, 80 ns and 100 ns, along with their corresponding (**B**) 2D interaction maps.

Table S1. Hydrogen bond pairs between monomers of 14-3-3σ in apo and GCP-Lys-OMe-bound systems at 100 ns simulation. Only the residues at H1-H4 (the dimer interface) are shown. Similar pairs (i.e. involving the same residues) between the two systems are bolded.

System	Pair ID	Donor - Acceptor	Atom number
Apo (14-3-3σ alone)	**1**	**84TYR(HH) – 21ASP(OD1)**	**3177 - 218**
	2	77LYS(HZ1) – 8GLN(OE1)	3100 - 77
	3	9LYS(HZ1) – 83GLU(OE2)	2425 - 825
	**4**	**9LYS(HZ1) – 83GLU(OE1)**	**2425 - 824**
	5	8GLN(E21) – 77LYS(O)	2413 - 770
	**6**	**84TYR(HH) – 21ASP(OD2)**	**843 - 2553**
	**7**	**18ARG(H21) – 91GLU(OE2)**	**181 - 3254**
	**8**	**18ARG(H11) – 55GLN(OE1)**	**178 - 2870**
Bound (14-3-3σ/GCP-Lys-OMe complex)	**1**	**84TYR(HH) – 21ASP(OD1)**	**3177 - 218**
	2	69SER(H) – 8GLN(OE1)	3021 - 77
	3	68LYS(HZ1) – 15GLN(OE1)	3015 - 146
	**4**	**18ARG(H21) – 91GLU(OE2)**	**2515 - 920**
	5	18ARG(H21) – 91GLU(OE1)	2515 - 919
	6	18ARG(H11) – 91GLU(OE1)	2512 - 919
	**7**	**18ARG(H11) – 55GLN(NE2)**	**2512 - 537**
	8	18ARG(HE) – 84TYR(OH)	2509 - 842
	**9**	**84TYR(HH) – 21ASP(OD1)**	**843 - 2552**
	10	11LYS(HZ1) – 70ASN(OD1)	110 - 3033
	**11**	**9LYS(HZ1) – 83GLU(OE1)**	**91 - 3158**
	12	8GLN(E21) – 80GLU(OE1)	79 - 3123
	13	8GLN(E21) – 68LYS(O)	79 - 3019

Table S2. Hydrogen bond occupancy between monomers of 14-3-3σ in apo system throughout the whole 100 ns simulation. Only the residues at H1-H4 (the dimer interface) are shown.

Pair ID	Donor - Acceptor	Atom number	Hydrogen bond occupancy (%)
1	84TYR(HH) – 21ASP(OD2)	843 - 2553	45.8
2	84TYR(HH) – 21ASP(OD1)	843 - 2552	39.8
3	84TYR(HH) – 21ASP(OD2)	3177 - 219	37.9
4	84TYR(HH) – 21ASP(OD1)	3177 - 218	37.9
5	9LYS(HZ1) – 83GLU(OE2)	91 - 3159	29.3
6	9LYS(HZ1) – 83GLU(OE1)	91 - 3158	28.1
7	80GLU(H) – 8GLN(OE1)	784 - 2411	23.5
8	18ARG(H11) – 84TYR(OH)	2512 - 842	20.1
9	18ARG(HE) – 84TYR(OH)	175 - 3176	19.9
10	9LYS(HZ1) – 83GLU(OE1)	2425 - 824	19.1
11	9LYS(HZ1) – 83GLU(OE2)	2425 - 825	17.6
12	18ARG(HE) – 84TYR(OH)	2509 - 842	17.5
13	18ARG(H11) – 55GLN(OE1)	2512 - 536	17.1
14	18ARG(H21) – 55GLN(OE1)	2515 - 536	15.6
15	18ARG(H11) – 55GLN(OE1)	178 - 2870	13.7
16	5SER(HG) – 80GLU(OE2)	2384 - 790	11.5
17	18ARG(H21) – 55GLN(OE1)	181 - 2870	10.5
18	5SER(HG) – 80GLU(OE1)	2384 - 789	10.1
19	8GLN(E21) – 74SER(O)	79 - 3071	10.0

Table S3. Hydrogen bond occupancy between monomers of 14-3-3σ in GCP-Lys-OMe-bound systems throughout the whole 100 ns simulation. Only the residues at H1-H4 (the dimer interface) are shown.

Pair ID	Donor - Acceptor	Atom number	Hydrogen bond occupancy (%)
1	84TYR(HH) – 21ASP(OD2)	3177 - 219	43.0
2	15GLN(E21) – 69SER(OG)	148 - 3024	36.9
3	84TYR(HH) – 21ASP(OD1)	843 - 2552	36.8
4	84TYR(HH) – 21ASP(OD2)	843 - 2553	35.9
5	84TYR(HH) – 21ASP(OD1)	3177 - 218	35.8
6	18ARG(H11) – 55GLN(OE1)	178 - 2870	28.8
7	9LYS(HZ1) – 83GLU(OE1)	2425 - 824	28.5
8	9LYS(HZ1) – 83GLU(OE2)	2425 - 825	27.5
9	9LYS(HZ1) – 83GLU(OE2)	91 - 3159	27.5
10	68LYS(HZ1) – 15GLN(OE1)	3015 - 146	26.1
11	9LYS(HZ1) – 83GLU(OE1)	91 - 3158	25.4
12	18ARG(H11) – 55GLN(OE1)	2512 - 536	24.2
13	18ARG(HE) – 84TYR(OH)	175 - 3176	19.8
14	18ARG(HE) – 84TYR(OH)	2509 - 842	15.8
15	70ASN(H) – 15GLN(OE1)	3029 - 146	14.1
16	69SER(H) – 8GLN(OE1)	3021 - 77	13.5
17	18ARG(H21) – 55GLN(OE1)	181 - 2870	12.4
18	8GLN(E21) – 68LYS(O)	79 - 3019	11.4
19	8GLN(E21) – 75GLU(O)	79 - 3081	11.1
20	18ARG(H11) – 84TYR(OH)	178 - 3176	11.1
21	11LYS(HZ1) – 71GLU(OE1)	110 - 3045	10.8
22	18ARG(H21) – 91GLU(OE1)	2515 - 919	10.3
23	11LYS(HZ1) – 71GLU(OE2)	110 - 3046	10.1

Table S4. Hydrogen bond occupancy between monomers of 14-3-3σ and GCP-Lys-OMe in GCP-Lys-OMe-bound systems throughout the whole 100 ns simulation. Only the residues at H1-H4 (the dimer interface) are shown.

Pair ID	Donor - Acceptor	Atom number	Hydrogen bond occupancy (%)
1	20GLU(H) – 234UNK(O20)	2538 - 4695	61.8
